# Porencephaly in an Italian neonate with foetal alcohol spectrum disorder: A case report

**DOI:** 10.1097/MD.0000000000021384

**Published:** 2020-07-31

**Authors:** Annalisa Mencarelli, Paolo Prontera, Gabriela Stangoni, Stefania Troiani, Tiziana Becchetti, Alessandra Pacitto, Susanna Esposito

**Affiliations:** a Pediatric Clinic, Department of Medical and Surgical Sciences, Università degli Studi di Perugia; b Medical Genetics Unit, Santa Maria della Misericordia Hospital; c Neonatology Unit, Santa Maria della Misericordia Hospital, Perugia; d Pediatric Clinic, Pietro Barilla Children's Hospital, Department of Medicine and Surgery, University of Parma, Parma, Italy.

**Keywords:** alcohol exposure, case report, ethanol, ethylglucuronide, foetal alcohol spectrum disorder, porencephaly

## Abstract

**Introduction:**

Foetal alcohol spectrum disorder (FASD) is a complex malformative disease caused by the teratogenic effect of alcohol consumed during pregnancy. Mothers are frequently reluctant to admit alcohol consumption during pregnancy. During infancy and particularly during neonatal period, differential diagnosis is difficult.

**Patient concerns:**

This case is represented by an Italian neonate boy small for gestational age, born by caesarean section at a gestational age of 37 weeks + 6 days by neglect and single-parent pregnancy. On physical examination, he presented particular facial features: microcephaly, epicanthal folds, flat midface, low nasal bridge, indistinct philtrum, and thin upper lip; moreover, examination revealed a macro-penis and recurvation without evidence of glans.

**Diagnosis:**

Echocardiogram showed an inter-ventricular defect of medium-muscular type and brain magnetic resonance imaging showed asymmetry of the cerebral hemispheres with hypoplasia of the left cerebral hemisphere, dilatation of the left ventricle, cerebrospinal fluid cavity, and porencephaly.

**Interventions:**

We investigated the ethylglucuronide (EtG) concentration in the neonate's hair by liquid chromatography-tandem mass spectrometry and we detected EtG in the infant's hair (normal value, 30 pg/mg), demonstrating prenatal alcohol exposure.

**Outcomes:**

In this neonate, EtG measure in hairs permitted the diagnosis of FASD, so allowing to exclude genetic diseases associated with similar clinical findings. After this result the mother admitted that she drunk alcohol during pregnancy (she declared 3 glasses of wine every day). At the age of 6 months, the child showed a moderate neurodevelopmental delay.

**Conclusion:**

This case shows that FAD should be considered in neonates with rare neurological diseases as porencephaly. In neonates and infants born to a mother who did not report alcohol use, EtG measure in hairs can significantly improve diagnosis of FASD, so allowing to exclude genetic diseases associated with similar clinical findings.

## 1 Introduction

Prenatal exposure to alcohol results in a number of somatic, cognitive and behavioral abnormalities that are defined as foetal alcohol spectrum disorders (FASD).^[1]^ In this group, according with the clinical phenotype and severity, are included the foetal alcohol syndrome (FAS), the partial FAS, the alcohol-related neurodevelopment disorder and alcohol-related birth defects. FAS is the most severe type of FASD as subjects with this syndrome have relevant facial abnormalities (short palpebral fissures, thin vermilion border of the upper lip, smooth filtrum), prenatal and/or postnatal growth deficiencies, deficient brain growth, abnormal morphogenesis, or abnormal neurophysiology, and neurobehavioral impairment.^[1]^ Subjects with less severe symptoms are considered partial FAS cases. Alcohol-related neurodevelopment disorder cases have mainly neurological manifestations, whereas patients with alcohol-related birth defects have one or more specific major malformations involving the cardiovascular system, the skeleton, the kidney and the urinary tract, the eye and the ear.^[1]^ Porencephaly is included among possible manifestations.

FASD cases are extremely common, although prevalence can significantly vary from geographic area to geographic area. The global prevalence among children and youth in the general population has been calculated to be 7.7 per 1000 population with the highest value in the World Health Organization (WHO) European Region (19.8), and the lowest in the World Health Organization (WHO) Eastern Mediterranean Region (0.1).^[2]^ Among countries, the highest values were found in South Africa, Croatia and Ireland with values of 111, 53.3, and 47.5 per 1000 population, respectively. However, it is highly likely that the prevalence rates are significantly higher than reported and that they can increase in the next few years.

Mothers are frequently reluctant to admit alcohol consumption during pregnancy. May et al reported that only one third of the mothers whose children were suffering from FASD had reported use of alcohol before diagnosis.^[3]^ Moreover, many cases are not diagnosed or are misdiagnosed. For example, many children with mild clinical manifestations are classified as attention deficit hyperactive disorders.^[4]^

The number of unplanned pregnancies is increasing^[5]^ as it is, at least in some countries, the rate of binge drinking and drinking among women of childbearing age.^[6]^ As FASD remains the most important cause of mental retardation and cause relevant medical, social and economic problems, early identification of FASD cases is critical. Despite no definitive therapy is available, several studies have shown that early interventions specifically planned to support family, solve neurological, behavioral and psychological problems, manage comorbid conditions, and assure nutritional support can significantly improve the quality of life of these patients.^[7]^

Unfortunately, identification of FASD cases frequently occurs several months or years after birth when interventions are too late to be significantly effective. Mean age of diagnosis is about 3 years and only 7% of the children are diagnosed in the first days of life.^[8]^ This because diagnosis of FASD is relatively easy in older children when signs and symptoms are definitively established and differentiation of FASD from other syndromes can be made taking in account only clinical manifestations. On the contrary, during infancy and particularly during neonatal period differential diagnosis is difficult and a different approach have to be followed if FASD have to be confirmed. The case here reported is a good example in this regard. In a neonate with FASD born to a mother who did not report alcohol use, diagnosis was made only after most of the genetic syndromes that have similar manifestations were excluded and a reliable test revealed presence of alcohol metabolites in the infant.

## 2 Case presentation

### 2.1 Presenting concerns

An Italian neonate boy small for gestational age, was born by caesarean section at a gestational age of 37 weeks + 6 days by neglect and single-parent pregnancy. The mother had denied having taken alcohol and drugs during pregnancy. Birth weight was 2300 g (30th percentile), his birth height was 45 cm (30th percentile), his birth head circumference was 29 cm (<30th percentile), and his Apgar score was 8/9 (1 minute/5 min). Only 1 prenatal ultrasound scan was given, and screenings for toxoplasma, rubella and cytomegalovirus infections were not performed. This case report was approved by the Ethics Committee of the Umbria Region (PED-2019-03) and written informed consent was obtained from both parents. Parents also signed the consent for the publication of this case report.

### 2.2 Clinical findings

**Figure 1:**
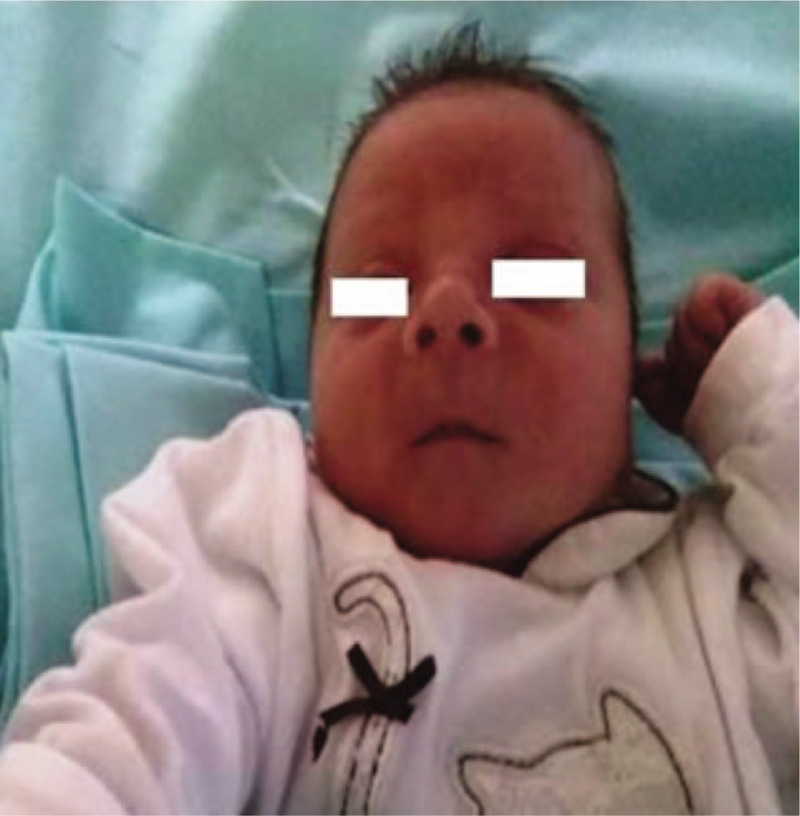
Dysmorphic features of the patient. Microcephaly, flat midface, indistinct philtrum, thin upper lip.

On physical examination, he presented particular facial features: microcephaly, epicanthal folds, flat midface, low nasal bridge, indistinct philtrum, and thin upper lip (Fig. 1); moreover, examination revealed a macro-penis and recurvation without evidence of glans.

### 2.3 Diagnostic focus and assessment

The initial investigation included blood cell count (white blood cells: 8270/μL; red blood cells: 4390,000/μL; Hb: 17.0 g/dL; platelets: 257,000/μL), biochemistry (SGOT: 57 U/L; SGPT: 15 U/L, Na: 133 mEq/L; K: 3.7 mEq/L; Cl 103 mEq/L; Ca: 5.20 mEq/L), thyroid function (TSH 5.3 mIU/L), metabolic tests and urine organ acid determination, all of which were within normal limits. Echocardiogram showed an inter-ventricular defect of medium-muscular type, normal aortic diameter, and no evidence of mitral valve prolapse. Abdomen ultrasonography was normal, brainstem response electric audiometry was bilaterally normal, and eye examination appeared without particular abnormalities.

**Figure 2:**
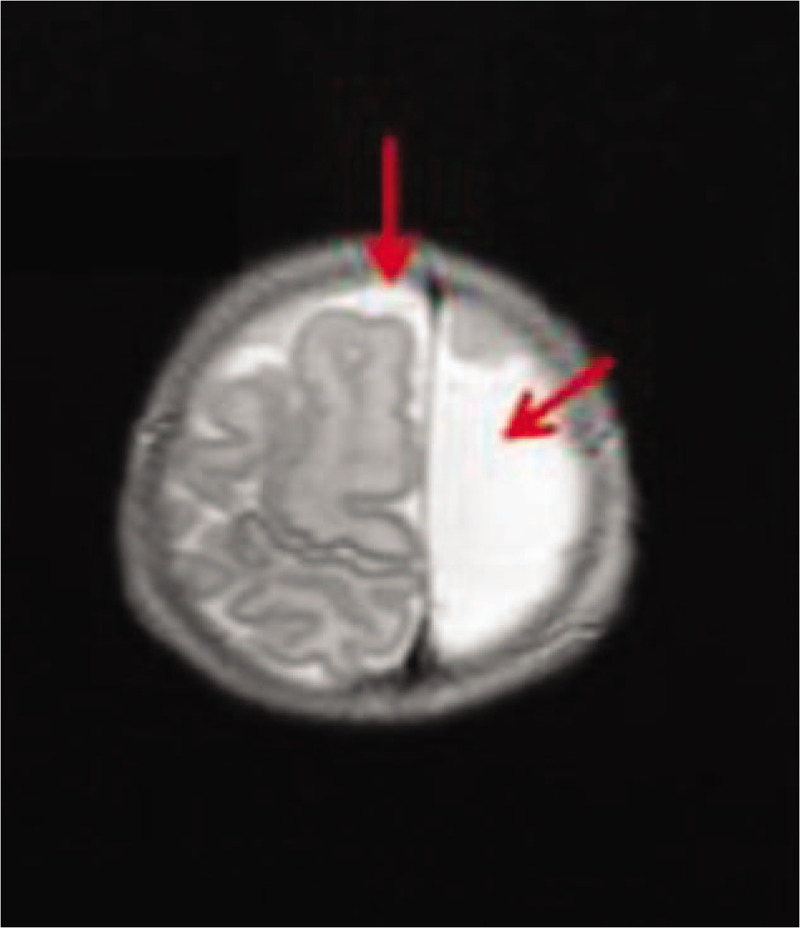
Brain magnetic resonance imaging. Asymmetry of cerebral hemispheres with hypoplasia of left cerebral hemisphere, dilatation of left ventricle (red flags) and porencephaly.

On the fourteenth day, brain magnetic resonance imaging (MRI) showed asymmetry of the cerebral hemispheres with hypoplasia of the left cerebral hemisphere, dilatation of the left ventricle, cerebrospinal fluid cavity, and porencephaly (Fig. 2). Porencephaly is a rare congenital disorder that results in cystic degeneration and encephalomalacia and the formation of porencephalic cysts. In this patient, MRI showed that porencephaly was located within the brain matter and employed the frontal lobe. The image represented a cavitated outcome of parenchymal damage with cortical dysgenesis occurring during intrauterine life. The cerebellar hemispheres and cerebellar vermis were normal.

### 2.4 Therapeutic focus and assessment

Conventional karyotyping revealed a normal male with 46, XY; Array-CGH (Array- Comparative Genomic Hybridization) genetic examination, performed in the presence of a complex brain malformation, was negative.

**Figure 3:**
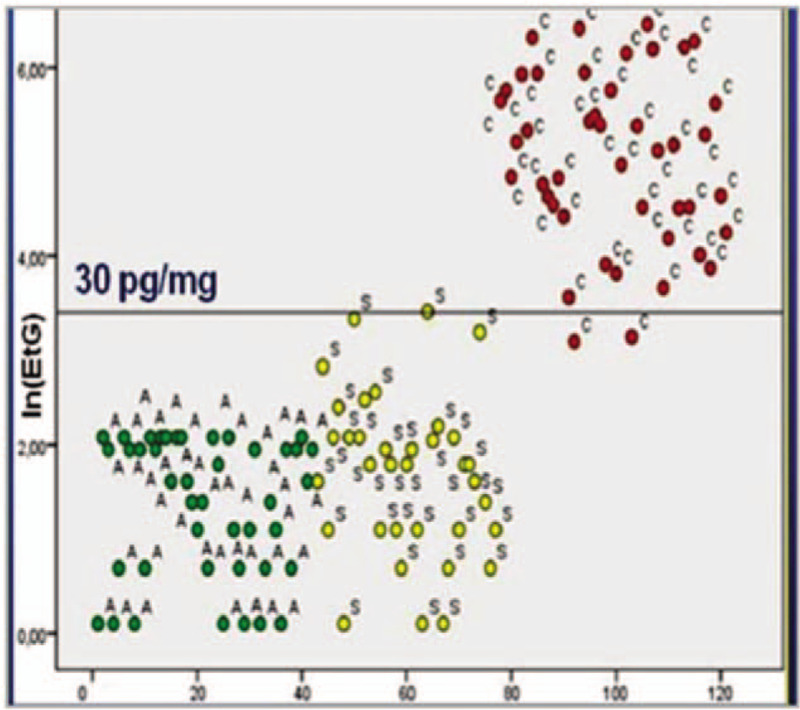
Liquid chromatography-tandem mass spectrometric. EtG concentration in neonatal hair (779.4 pg/mg) shows intrauterine exposure to ethanol. The cut-off value was 30 pg/mg.

To exclude or confirm FASD, the ethylglucuronide (EtG) concentration in the neonate's hair was evaluated. Six centimeters of proximal hair segments (weight ranging from 60 to 80 mg) from our neonate were tested; EtG was measured by liquid chromatography-tandem mass spectrometry according to the method described by Morini et al.^[9]^ EtG (ie, a biomarker of in utero exposure to ethanol intake during pregnancy) in neonatal hair was positive, showing high levels (779.4 pg/mg) of more than 20 times the cut-off value established for all the age groups (30 pg/mg) (Fig. 3).

### 2.5 Follow-up and outcomes

In this neonate, EtG measure in hairs permitted the diagnosis of FASD, so allowing to exclude genetic diseases associated with similar clinical findings. After this result the mother admitted that she drunk alcohol during pregnancy (she declared 3 glasses of wine every day). At the age of 6 months, the child showed a moderate neurodevelopmental delay. The status of porencephalic cyst at MRI was unchanged.

## 3 Discussion

This case shows a particular phenotype in an Italian infant with a rare brain malformation (ie, porencephaly), expanding our knowledge on the spectrum of FAD.

FASD is the leading cause of mental retardation and carries a significant medical, social and economic burden.^[1]^ This explains why interventions to abolish alcohol consumption by pregnant women have been made by several national health organizations worldwide.^[10]^ Moreover, to favor early identification of FASD and assure effective interventions several diagnostic guidelines have been prepared.^[11]^ However, they are effective only in toddlers and older children. In neonates and young infants they are not adequate and diagnosis of FASD can be made only through a number of additional intervention able to exclude the conditions that can mimic FASD.^[11]^ Accurate analysis of maternal conditions, including use of drugs toxic for the fetus, must be made. Moreover, a number of genetic conditions have to be taken into account.^[11]^ Finally, even if the mother does not report alcohol use, laboratory tests able to identify alcohol metabolites in the mother or in the fetus must be performed.^[11]^

A complex diagnostic approach is needed because neonates born to mothers that did not receive alcohol but other embryotoxic factors such as valproic acid, hydantoin, toluene can have facial abnormalities, low birth weight, and developmental delays that can mimic FASD.^[1,12]^ Mothers with phenylketonuria that receive unproper diets and develop very high blood levels of phenylalanine can generate children with microcephaly, craniofacial abnormalities, poor intrauterine growth, and neurological developmental delay. Abuse of cocaine or other illegal drugs can lead to similar clinical features. Moreover, a variety of genetic abnormalities enter in the differential diagnosis. Aarskod syndrome, Bloom syndrome, Cornelia de Lange syndrome, Dubowitz syndrome, Noonan syndrome, Williams syndrome and velocardiofacial syndrome have some clinical characteristics that can be initially confused with FASD.^[1,12]^ Genetic studies are essential to confirm or exclude these conditions.

Prenatal alcohol exposure interferes with normal neurological development through a range of mechanisms including cortical dysgenesis as porencephaly.^[13]^ Porencephaly can occur as a result of alcohol consumption during pregnancy, indeed ethanol can induce toxic brain injury, or ischemia or hemorrhage. Porencephalic cysts are an uncommon congenital finding. The cysts are typically lined by white matter. They are thought to occur from focal encephalomalacia during early gestation.^[13]^ With MRI as with computed tomography, the cysts appear well defined and often correspond to a vascular territory.^[14]^ Importantly the cysts are not lined by grey matter, helpful in distinguishing them from arachnoid cysts and schizencephaly. Typically the cysts are seen to communicate with the ventricles and/or the subarachnoid space. Porencephaly management is essentially supportive.^[14]^

The knowledge of the main mechanisms of brain alcohol damage is important to improve the care and the prevention of complications of effects in utero ethanol exposure. A fundamental role for a proper and early diagnosis of FASD is played by the analysis of ethanol metabolites in the biological tissues. Most of the studies in this regard have used maternal tissues. However, the evidence of alcohol metabolites in the child is a direct evidence of the strict relationships between alcohol ingestion by the mother and the development of FASD, particularly when the woman did not report alcohol consumption. In this case, the association between use of toxic drugs by the mother was considered very unlikely as the woman did not suffer from diseases treated with drugs and no opiates were found in her urine. Most of the genetic syndrome that mimic FASD were excluded by genetic analysis. Finally, despite clinical findings strongly suggested FASD, diagnosis was definitively made only when alcohol metabolites in the child hairs were studied. EtG was measured in neonate's hair as this compound is considered a reliable biomarker of alcohol exposure in women who did not disclose their alcohol use, although did not correspond to alcohol use in very early pregnancy.^[9]^ Moreover, neonate's hairs were tested because they offer a very long window of detection. Compared to other markers, EtG has higher diagnostic sensitivity and specificity and when measured in hairs can show alcohol intake >60 g/day in the previous 3 to 6 months. It is a product of nonoxidative ethanol metabolism and, contrarily to the compounds derived by liver metabolization remains in circulation somewhat longer and is incorporate stably into hair upon repeated drinking.^[15]^

## 4 Conclusion

This case shows that FAD should be considered in neonates with rare neurological diseases as porencephaly. In neonates and infants born to a mother who did not report alcohol use, EtG measure in hairs can significantly improve diagnosis of FASD, so allowing to exclude genetic diseases associated with similar clinical findings. Further studies are needed to improve the care and the prevention of complications of effects of in utero alcohol exposure.

## Acknowledgment

We would like to thank all the pediatricians, geneticists, and nurses involved in the management of this child, as well as the patient's parents.

## Author contributions

AM wrote the first draft of the manuscript and participated in patient's management; PP and GS performed the diagnosis; ST and TB were in charge for clinical management; AP performed the literature review; SE revised the manuscript and gave a substantial scientific contribution. All the authors read and approved the final version of the manuscript.
